# Infective Endocarditis Presented as a Right Atrium Mass in a Patient with Ulcerative Colitis

**DOI:** 10.1155/2015/789170

**Published:** 2015-05-24

**Authors:** Ali Asghar Moeinipour, Hamid Hoseinikhah, Mohamad Ali Kiani, Naser Tayyebi Meibodi, Mohammad Hassan Aelami, Ali Reza Sepehri Shamloo, Shahram Amini

**Affiliations:** ^1^Atherosclerosis Prevention Research Center, Imam Reza Hospital, Mashhad University of Medical Sciences, Mashhad 9137913316, Iran; ^2^Quaem Hospital, Mashhad University of Medical Sciences, Mashhad 9137913316, Iran; ^3^Imam Reza Hospital, Mashhad University of Medical Sciences, Mashhad 9137913316, Iran

## Abstract

Involvement of the heart is infrequently seen in irritable bowel syndrome (IBD). We present a case of severe acute infective endocarditis diagnosed as ulcerative colitis in further workup.

## 1. Introduction

Irritable bowel syndrome (IBD) is a disease commonly encountered in modern society with increasing frequency in developed countries. It can promote IE (infective endocarditis) by increasing transmucosal permeability and thus facilitating bacterial invasion to the blood stream [1–4]. Moreover, the need for immunosuppressive drugs and use of invasive procedures and devices such as CVC (central venous catheter) are additional predisposing factors. Cardiovascular involvement in IBD has occasionally been reported mainly in the form of case reports [5–7]. Endocardium derangement in IBD includes IE and subendocardial abscess. The causative agent of IE in IBD may be bacterial or fungal and may require surgery. An increased risk of endocarditis has been suggested in IBD patients but it has been reported in less than 30 cases [3, 6]. In one study, among 213 consecutive patients treated for proven native valve endocarditis, six (2.8%) had inflammatory bowel diseases (three with ulcerative colitis and three with Cohn's disease) [8].

Bacteremia during disease exacerbation seems to be the most probable pathophysiological mechanism of this complication [6–9].

Here we present a child presenting with chest pain, dyspnea, and active rectal bleeding diagnosed as infective endocarditic and ulcerative colitis.

## 2. Case Report

**Figure 1:**
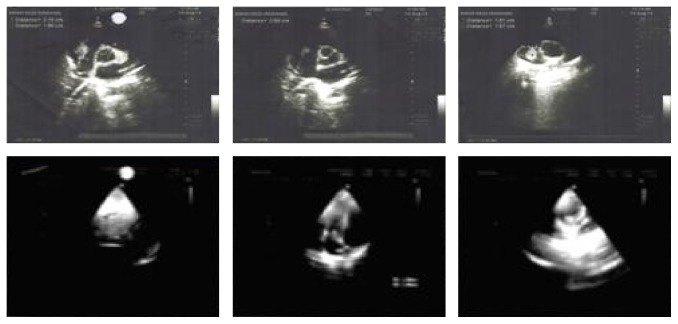
Transesophageal echocardiography (TEE) of a right atrial mass in the patient.

A 12-year-old female was admitted to pediatric ward with rectorrhagia, dyspnea, chest pain, diarrhea, and fever. During the evaluation of dyspnea and chest pain with transthoracic echocardiography, a large mobile mass was visualized in lateral wall of right atrium (RA) diagnosed as atrial myxoma (Figure 1). Chest radiography was without any pathologic finding.

The patient underwent operation using bicaval occlusion method without cardiopulmonary bypass (CPB) [9]. After opening RA, a mass was removed from lateral wall of RA. After revision of the tricuspid valve and other sites of RA and RV and confirmation by intraoperative TEE, the sternum was closed and the patient was sent to ICU. She got extubated after 5 hours and 30 minutes and left ICU uneventfully after 3 days. She was referred to the gastrointestinal service for evaluation and management of her gastrointestinal symptoms with no cardiovascular symptoms after 3 days of stay in the postcardiac surgery ward.

**Figure 2:**
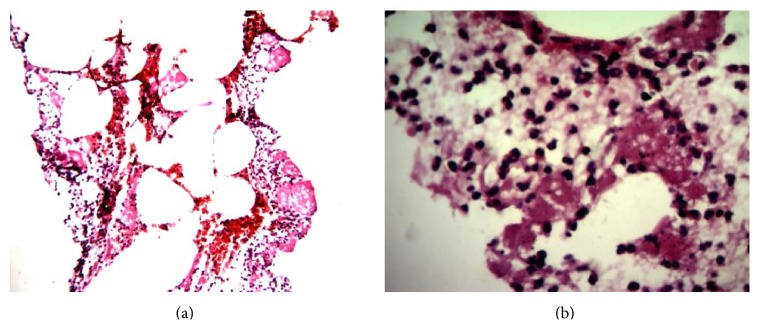
Microscopic examination of right atrial mass in the patient.

Microbiologic and pathologic examination of RA mass revealed acute inflammation (Figures 2(a) and 2(b)) with isolation of* Enterobacter aerogenes*. She received intravenous antibiotics including ceftazidime and vancomycin. Blood culture also showed the same organism.

In the pediatric ward, she underwent colonoscopy and acute inflammation was visualized in the entire colorectal area. Pathologic examination reported active ulcerative colitis for which she received appropriate treatment including metronidazole and corticosteroid. Her pulmonary symptoms resolved four days after the operation and she was discharged home after 15 days with good clinical status. Her follow-up after 1, 3, and 6 months revealed no cardiopulmonary symptoms and only occasional mild abdominal pain.

## 3. Discussion

IBD, whose incidence and prevalence are increasing worldwide, can promote IE by increasing transmucosal permeability and thus facilitating bacterial invasion to the bloodstream predisposing IBD patients to IE. An increased risk of endocarditis has been suggested in IBD patients but it has been reported in less than 30 cases [7].

Bacteremia during disease exacerbation seems to be the most probable pathophysiological mechanism of this complication. Prophylaxis for bacterial endocarditis should be carefully considered before expected bacteremia in patients with highly active inflammatory bowel disease even in the absence of cardiac factors predisposing to bacterial endocarditis [7, 9]. It usually presents during disease relapse or it is the presenting symptom of an undiagnosed quiescent ulcerative colitis [6, 9]. It usually affects aortic and mitral valve that may require surgery [9, 10].Our case presented with dyspnea and chest pain with mild gastrointestinal (GI) symptoms including rectal bleeding and diarrhea. Her family had not sought medical advice for her gastrointestinal symptoms until she developed chest pain and dyspnea. Her ulcerative colitis was diagnosed in the hospital after GI workup. It seems that IBD has been a predisposing factor for IE in this case. We assumed that dyspnea and chest pain can be attributed to endocarditic and septic condition in this patient that resolved after surgery. Furthermore, unlike most other studies we have shown that ulcerative colitis can be associated with infective endocarditis of the right side of the heart as well.

## 4. Conclusion

Although heart involvement in IBD is rare, every clinician must be aware of these extraintestinal complications especially if the patient presents with cardiopulmonary symptoms.
